# Supplementary material for: Biological effects related to exposure to polychlorinated biphenyl (PCB) and decabromodiphenyl ether (BDE-209) on cats
Source: PLoS One. 2023 Jan 20;18(1):e0277689. doi: 10.1371/journal.pone.0277689 (PMC9858064; doi:10.1371/journal.pone.0277689)
Supplement: Attachment — Submitted filename: 2. Response to Reviewers.docx [file pone.0277689.s007.docx]

Dear Dr. Anjani Kumar Tiwari

Submission no: PONE-D-22-09902

Submission title: Biological effects of polychlorinated biphenyl (PCB) and decabromodiphenyl ether (BDE-209) exposure on cats

Thank you very much for your letter dated 10 Jun 2022 with the reviewer’s comments.

The following are our responses to reviewer’s comments regarding this manuscript. The revised words or sentences have been highlight in yellow.

ACADEMIC EDITOR:

The language of this manuscript is not very appropriate therefore author should rewrite and recheck it.

This manuscript was revised and checked by an experienced editor whose first language is English.

Reviewers' comments:

Reviewer #1:

1.As the authors claim that this is the first study to provide information on the biochemical effects of organohalogen compounds, so some more relevant studies along with risk assessment study is important. Highlight on that part.

It was revised and highlighted on the parts of the abstract, introduction, and discussion. (Page 2, Line 41-44, Page 4, Line 65-69, and Page 21-22, Line 407-409) In addition, references related to the risk assessment of OHCs are shown below;

Ma et al., 2017. Environmental Research. 2017;155:116-22.

Mizukawa et al., Environ Sci Technol. 2016;50(1):444-52.

Zheng et al., 2015. Environment International. 2015;78:1-7.

Shoeib et al., 2012. Environmental Pollution. 2012;169:175-82.

Nomiyama et al., 2022. Science of The Total Environment. 2022;842:156490.

Walter et al., 2017. BMC Vet Res. 2017;13(1):120.

Mensching et al., 2012. J Toxicol Environ Health A. 2012;75(4):201-12.

Norrgran et al., 2015. Environ Sci Technol. 2015;49(8):5107-14.

2.Is it possible to study the biochemical effects with other organohalogen compounds (OHC) in cats (instead of PCBs and PBDEs)? Enlightenment is required.

It’s possible to study with other organohalogen compounds such as DDTs, but in this study, we conducted this experiment with exposure to the PCBs and BDE-209 because there are several biomonitoring studies that detected PCBs and PBDEs have been commonly found in the blood of pet cats, and indicated health effects such as hyperthyroidism and type 2 diabetes mellitus. On the other hand, it has already been reported that the major DDT metabolite, p,p*'*-DDE, was the major OHC in Pakistani cat serum (N=20) and ranged between 1 and 2150 ng/g lipid weight (Ali et al., 2013, Sci Total Environ, 449:29-36), suggesting it needs to monitoring and risk assessment for other OHCs in future. This reason is highlighted in the parts of introduction (Page 3-4, Line 64-68) and discussion (Page 17, Line 301-312).

3.Some current references (after the year: 2019) are required to the relevant field.

Since the studies of PCB and PBDE residues in pet cats are limited recently (year: 2020-2022), these below references related to our study were added to this manuscript:

Mizukawa H, Nomiyama K. Biotransformation of Brominated Compounds by Pet Dogs and Cats. In: Pastorinho MR, Sousa ACA, editors. Pets as Sentinels, Forecasters and Promoters of Human Health. Cham: Springer International Publishing; 2020. p. 107-21.

Pastorinho M, Sousa ACA. Pets as Sentinels, Forecasters and Promoters of Human Health. Pets as Sentinels, Forecasters and Promoters of Human Health. 2020.

Nomiyama K, Yamamoto Y, Eguchi A, Nishikawa H, Mizukawa H, Yokoyama N, et al. Health impact assessment of pet cats caused by organohalogen contaminants by serum metabolomics and thyroid hormone analysis. Science of The Total Environment. 2022;842:156490. doi: https://doi.org/10.1016/j.scitotenv.2022.156490.
